# Antibodies against C1q Are a Valuable Serological Marker for Identification of Systemic Lupus Erythematosus Patients with Active Lupus Nephritis

**DOI:** 10.1155/2015/450351

**Published:** 2015-10-13

**Authors:** Shuhong Chi, Yunxia Yu, Juan Shi, Yurong Zhang, Jijuan Yang, Lijuan Yang, Xiaoming Liu

**Affiliations:** ^1^Department of Rheumatology, The General Hospital of Ningxia Medical University, Yinchuan 750004, China; ^2^Center of Laboratory Medicine, The General Hospital of Ningxia Medical University, Yinchuan 750004, China; ^3^Institute of Human Stem Cell Research, The General Hospital of Ningxia Medical University, Yinchuan, Ningxia 750004, China

## Abstract

*Objective*. An early diagnosis of lupus nephritis (LN) has an important clinical implication in guiding treatments of systemic lupus erythematosus (SLE) in clinical settings. In this study, the diagnostic values of circulating autoantibodies to C1q alone or in combination with other markers for accessing active SLE and LN were evaluated. *Methods*. The diagnostic value of anti-C1q autoantibodies for identification of patients with active SLE disease and LN was evaluated by analyzing the level of anti-C1q antibodies in sera from 95 SLE patients, 40 non-SLE patients, and 34 healthy cohorts. *Results*. The prevalence of anti-C1q antibodies was significantly higher in patients with SLE (50/95, 52.6%), active SLE (40/51, 78.4%), and LN (30/35, 85.7%) in comparison with non-SLE patient controls, patients with inactive SLE, and non-LN, respectively. A combination of anti-C1q with anti-dsDNA and/or levels of complements C3 and C4 exhibited an increased specificity but a decreased sensitivity for identification of patients with active SLE and LN diseases relative to each of these markers alone. *Conclusion*. Anti-C1q antibodies were strongly associated with disease activity and LN in SLE patients, suggesting that it may be a reliable serological marker for identification of SLE patients with active LN and active SLE disease.

## 1. Introduction

Systemic lupus erythematosus (SLE) is a chronic autoimmune disease with unknown etiology which can be characterized by producing various autoantibodies against self-antigens (autoantigens) [1]. SLE can affect multiple systems and major organs, among which lupus nephritis (LN) is a common major organ manifestation and a main cause of the morbidity and mortality of the disease [2]. In this regard, LN affects 40–80% of SLE patients, and an immunosuppressive treatment for LN may have an adverse effect on kidney and result in chronic renal failure, which sequentially increases the morbidity and mortality in SLE patients [1]. Therefore, an involvement of renal disease activity is one of the most important prognostic factors for patients with SLE, and the diagnosis of SLE patients with LN has an important clinical implication in guiding the treatment of SLE in clinical settings [3].

The aberrant production of a broad heterogeneous group of autoantibodies is a serological hallmark in SLE; a great effort has thus been made to understand the pathogenic, diagnostic, and prognostic value of these autoantibodies since they were discovered [2–4]. An evaluation of clinical relevance of the autoantibody profile and disease parameters will aid in identifying SLE patients at risk for specific complications at an early stage and thus enable clinicians to initiate an effective therapeutic strategy that possibly decreases the morbidity and mortality for patients with SLE [5].

There are more than 160 autoantibodies which have been reported in SLE patients, including antibodies to complement C1q, histone, chromatin, and nuclear and double-stranded DNA (dsDNA) [6]. Among these autoantibodies, anti-dsDNA and anti-C1q antibodies exhibited a stronger association with clinical features of active SLE particularly with the renal disease activity than other serological antibodies, indicating that they are an important value of measuring these autoantibodies in SLE patients [4]. Indeed, SLE patients with both anti-dsDNA and anti-C1q antibodies often had a manifestation of renal disease and poor renal outcome. In this regard, SLE patients with anti-C1q antibodies showed higher renal disease activity in comparison with those who did not have circulating anti-C1q antibodies [7]. This notion is supported by solid evidence that serum levels of complement C1q and anti-C1q autoantibodies are often decreased and elevated in patients with active LN, respectively [8]. Thus, circulating autoantibodies against C1q are considered as a biomarker for LN to predict renal injury and disease activity in SLE [8–18].

Complement C1q is a cationic glycoprotein produced by immune cells (macrophages, monocytes, and dendritic cells), fibroblasts, and epithelial cells, which is the first component identified in the canonical pathway of complement activation, with functions mainly to clear autoantigens generated during cell apoptosis and immune complexes from tissues [1, 19]. Antibodies against C1q (anti-C1q) were first reported in the serum of SLE patients in 1984 [20]. The prevalence of anti-C1q antibodies is ranged from 34% to 47% in patients with SLE [11, 12, 21, 22], but is 100% in patients with hypocomplementemic urticarial vasculitis syndrome (HUVS) [23]. In addition to C1q complement, serum levels of complements C3 and C4 are also decreased in SLE patients [24]. Therefore, levels of C1q, C3, and C4 and/or the autoantibodies to C1q, dsDNA, and chromatin/nucleosome in serum are important immunological markers in the diagnosis of SLE, particularly for LN disease [7, 10, 12, 13, 24–26].

In general, elevated autoantibodies against nucleosome and dsDNA (anti-dsDNA) and reduced complements were reported to correlate with disease activity and induction of glomerular inflammation in SLE [2, 5–7, 24–26, 28]; particularly anti-dsDNA antibodies exhibited a pathogenic importance in LN [7, 16]. However, antibodies to dsDNA and the reduction of complements were also found in non-LN patients and clinically inactive SLE patients with a relatively high percentage [29]. Such a lack of specificity of anti-dsDNA antibodies for renal flare was also observed in other conventional parameters such as anti-nucleosome antibody (ANA), levels of complements C3 and C4, proteinuria, and urine sediment [30], which led to searching other reliable immunological markers for identifying those SLE patients with active nephritis [16]. Among these markers, anti-C1q antibodies have been extensively studied in the diagnosis of a renal flare either alone or in combination with other serological markers, owing to the prevalence of anti-C1q in patients with active LN [7, 10–13, 15, 18, 22, 24, 26].

Several lines of evidence have shown a combination of anti-C1q, anti-dsDNA, and/or nucleosome antibodies was strongly correlated with renal flares and could be used for prognosis of patients with LN [7, 12]. Furthermore, anti-C1q antibodies have been suggested to correlate more strongly with renal flares than other serological markers [18]. In addition, patients with no anti-C1q antibodies were less likely to have active renal diseases [10, 12, 16]. Although a clinical association between the presence of anti-C1q and LN has been widely evidenced, the diagnostic value of anti-C1q antibodies in LN remains controversial and debatable, since anti-C1q antibodies were also found in healthy individuals [31]. In addition, available data for analyzing a correlation of anti-C1q antibodies with renal pathological characteristics and other clinical parameters for renal flares in SLE patients are limited. Moreover, there is no consensus on whether the anti-C1q antibodies are correlated with one type of LN or useful in the follow-up of patients with active LN [10, 13, 15, 26]. Therefore, there is a need to further evaluate diagnostic value of anti-C1q in clinical settings.

In the present report, we aimed to evaluate circulating anti-C1q antibodies of 95 SLE patients in a single center and investigate the clinical significance of such antibodies alone or in combination with anti-dsDNA antibodies and/or serum levels of C3 and C4 for accessing active nephritis in SLE patients. Our results suggest that serum anti-C1q antibody is more strongly associated with renal disease activity in SLE patients than anti-dsDNA antibodies and reduction of C3 and C4 in terms of diagnostic specificity. Furthermore, a combination of serum antibodies to C1q and dsDNA antibodies and the levels of C3 and C4 can increase the specificity of diagnosis for active LN.

## 2. Materials and Methods

### 2.1. Ethics Statement

Human blood samples were collected with a protocol approved by the Ethics Committee for the Conduct of Human Research at Ningxia Medical University (NXMU-E2012-102p). Written consent was obtained from every individual according to the Ethics Committee for the Conduct of Human Research protocol. For the participants younger than 18 years of age, written informed consents were obtained from their guardians or parents on behalf of the children. No special informed consent was required for Chinese Hui minority in this study. All participants were provided a written informed consent for the publication of the data. The PI of this study maintains human research records, including signed and dated consent documents, for ten years after the age of majority. The Ethics Committee for the Conduct of Human Research at Ningxia Medical University approved the consent procedure for this study (NXMU-2012-102e).

### 2.2. Serum Samples

Serum samples of 95 consecutive SLE patient samples (86 females and 9 males) were collected from the outpatient rheumatology clinics of the General Hospital of Ningxia Medical University from January 2011 to June 2012. The mean ± SD age for the SLE patients at the time the sample was drawn was 32.56 ± 11.37 years (range 14 to 71), with an average duration of diseases of 5.32 ± 4.04 (0.2 to 18 years). The American College of Rheumatology (ACR) criteria were used to diagnose a patient with SLE [32, 33], and the disease activity was defined according to SLE Disease Activity Index (SLEDAI) criteria [34, 35]. A patient with SLEDAI ≥10 was defined as active SLE. Renal involvement was defined based on clinical and laboratory manifestations. An active LN was defined as urine protein excretion ≥500 mg/day or cellular casts [32]. Sera of 40 gender- and age-matched patients with non-SLE autoimmune diseases (4 males and 36 females) were also collected; these included rheumatoid arthritis (RA) (8 cases), primary Sjögren's syndrome (pSS) (10 cases), ankylosing spondylitis (AS) (2 cases), dermatomyositis (DM) (5 cases), Behçet's disease (BD) (4 cases), systemic sclerosis (SSc) (3 cases), undifferentiated connective tissue disease (UCTD) (5 cases), and systemic vasculitis (3 cases). All cases were diagnosed with respective diagnostic criteria. The mean ± SD age for these non-SLE patients at the time the sample was drawn was 33.56 ± 11.87 years (range 15 to 69), with an average duration of diseases of 6.01 ± 3.79 (0.3 to 20 years). In addition, sera of 34 gender- and age-matched healthy individuals (3 males and 31 females) were also collected. These healthy control cohorts were recruited from those who had undergone comprehensive medical screening at the General Hospital of Ningxia Medical University and who had no history of chronic diseases and no family history of autoimmune diseases. The demographics of individuals involved in this study were outlined in Table 1. All sera were treated with heparin and frozen in 100 *μ*L aliquots at −80°C until analyzed. The ethnic populations of subjects in this study included Chinese Han and Chinese Hui, which were based on two criteria: they were of purely Han or Hui descents for at least three generations and individual ancestors have lived in the Ningxia region for at least three generations (Table 1). There was no genetic relationship among these individuals. All the samples were collected under informed consent.

### 2.3. Detection of Anti-C1q IgG Autoantibodies

The concentration of anti-C1q antibody in serum was measured by an enzyme-linked immunosorbent assay (ELISA) using commercially available kits according to the manufacturer's instruction (INOVA Diagnostics Inc., San Diego, CA, USA). Briefly, sera were diluted 1/100 and then added into each well; the wells were washed with high ionic strength buffer after being incubated at room temperature for 1 h. Then horseradish peroxidase coupled to anti-human IgG conjugate supplied with the kit was used as the secondary antibody. After incubation for 30 min, the wells were extensively washed for three times, followed by the addition of 100 *μ*L trimethylbenzene solution and incubation for 30 min before 100 *μ*L of stopping solution was added into each well. The optical density was then measured at 450 nm. The absorbance (OD_450 nm_) was then converted into a concentration through standard curve with a cutoff value of 10 AU/mL (determined by the manufacturer). Values less than 10 AU/mL were considered as negatives, and values equal to or greater than 10 AU/mL were considered as positives as suggested by the manufacturer. Other laboratory data, including urinalysis, serum levels of complements C3 and C4 and hemoglobin, anti-nuclear antibodies (ANA), anti-dsDNA antibodies, anti-ribonucleoprotein, perinuclear anti-neutrophil cytoplasmic antibody (pANCA), Sjögren's syndrome A (SSA) and B (SSB) antibodies, and anti-Smith (Sm), were also recorded, respectively. The sensitivity, specificity, and predictive values were calculated using formula described in a previous report [27].

### 2.4. Statistical Analysis

All laboratory data were entered into and extracted from PRISM (version 5) (GraphPad Software, La Jolla, CA, USA) and/or SPSS for Windows (version 17.0) (SPSS Inc., Chicago, IL, USA). Statistical evaluation of the data was performed by one-way ANOVA when more than two groups were compared with a single control and* t*-test for comparison of differences between the two groups. ROC (receiver operator characteristic) curve was used to find out the best cut of value and validity of certain variable. The association between qualitative variables was evaluated by Yates' *χ* ^2^ with correction or Fisher's exact test. Data were presented as the mean ± standard deviation (SD). A *p* value of less than 0.05 was considered as statistically significant.

## 3. Results

### 3.1. SLE Demographics Data

The unselected SLE population studied in this study included 86 (90.5%) females and 9 males (9.5%) with a mean age of 32.56 ± 11.37 years (range 14 to 71), and the average duration of diseases was 5.32 ± 4.04 (0.2 to 18 years). The mean of SLEDAI score of SLE was 12.03 ± 6.45 (range 0 to 35). The distribution of ethnic population was 79.0% of Chinese Han and 21.0% of Chinese Hui (Table 1). The demographics data of controls for patients with non-SLE autoimmune diseases and healthy subjects were also presented in Table 1.

### 3.2. Prevalence of Anti-C1q Antibodies and Correlation with SLE Disease Activity

**Figure 1:**
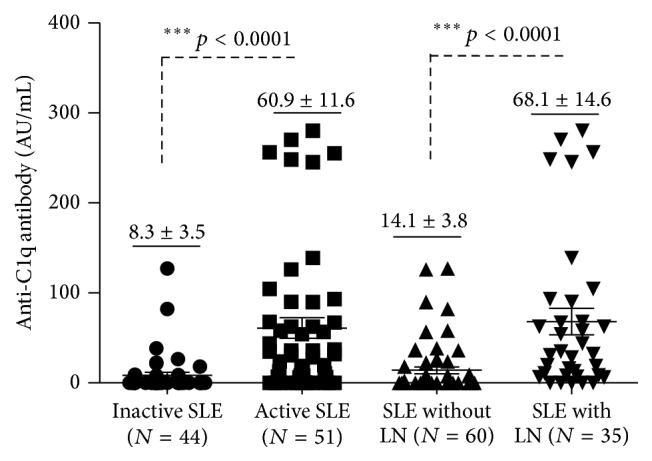
Differences in anti-C1q antibody levels based on SLE activity and LN activity in SLE patients. Bars indicate the average levels of anti-C1q antibodies in each group. Compared with the respective inactive and non-LN groups, ^*∗∗∗*^ *p* < 0.0001. Data present as the mean ± SD in each group.

**Figure 2:**
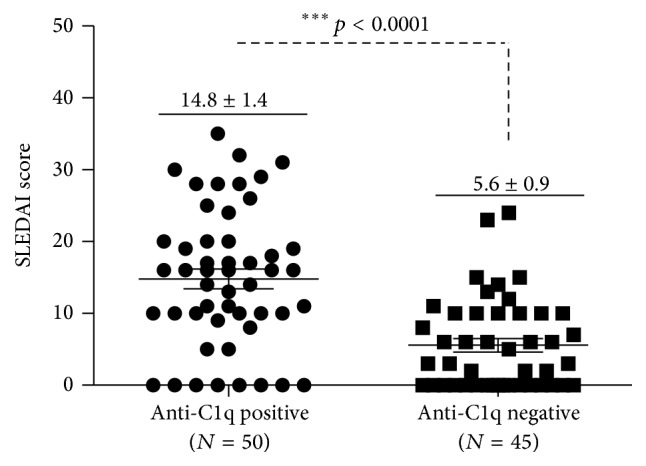
The SLEDAI score in anti-C1q positive and negative groups of SLE patients. Bars indicate the average SLEDAI score in each group. Compared with the respective anti-C1q negative group, ^*∗∗∗*^ *p* < 0.0001. Data present as the mean ± SD in each group.

**Figure 3:**
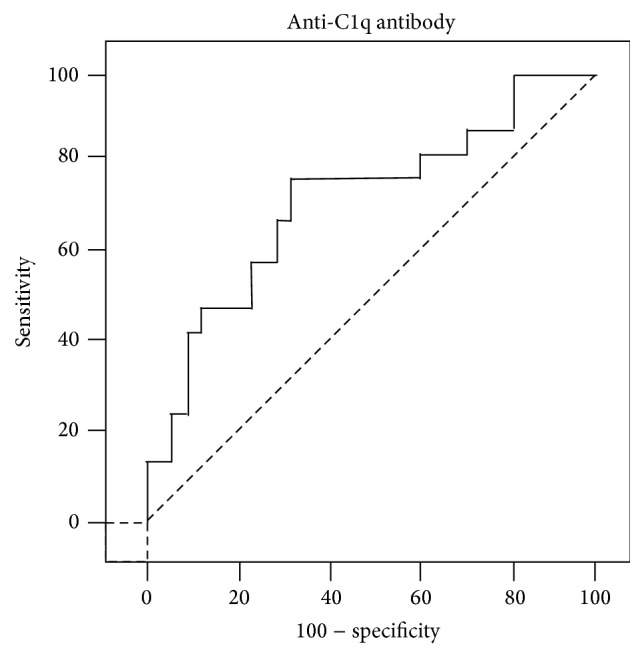
ROC curve for anti-C1q antibodies in active lupus nephritis.

Serum antibodies to C1q were determined in 50 of the 95 SLE patients (52.6%), which was consistent with finding from other studies [12, 36]. Patients with active SLE were more frequent (40/51, 78.4%) to have anti-C1q than those with inactive SLE (10/44, 22.7%) (Table 2). Importantly, anti-C1q antibodies were strikingly more often to be detected in the sera of LN patients (30/35, 85.7%) than in sera of those without a renal flare (20/60, 33.3%) (Table 3). More importantly, the concentration of anti-C1q antibodies was significantly higher in active SLE patients than in inactive SLE patients (60.9 ± 11.6 AU/mL versus 8.3 ± 3.5 AU/mL, *p* < 0.0001) (Figure 1 and Table 3). Of interest, a statistically higher titer of anti-C1q was also found in patients with LN relative to those SLE patients without a renal involvement (68.1 ± 14.6 AU/mL versus 14.1 ± 3.8 AU/mL, *p* < 0.0001) (Figure 1 and Table 3). Despite the fact that there was no significant difference found in distributions of gender (male/female), ethnicity, age, and disease duration between anti-C1q positive and negative groups (Table 2 and data not shown), the mean of SLEDAI score was higher in patients with positive anti-C1q antibodies (14.8 ± 1.4 versus 5.6 ± 0.9, *p* < 0.001) (Figure 2). The ROC curve also showed that anti-C1q antibodies were considered as better positive marker than negative in LN with higher sensitivity (Figure 3).

### 3.3. Significances of Anti-dsDNA Antibodies and Levels of C3 and C4 in SLE Disease Activity

Anti-nuclear antibodies (ANA) and dsDNA antibodies were the most prevalent autoantibodies observed in these SLE cohorts as determined by ELISA, which were detected in 100% (95/95) and 82.1% (78/95) of SLE patients, respectively (Table 3). In line with the frequency of anti-C1q antibodies detected in SLE, more frequent anti-dsDNA was detected in patients with active SLE or LN as compared with those with inactive SLE or without a renal involvement (Table 3). The titer of anti-dsDNA antibodies was also higher in the active SLE and LN groups in comparison with the inactive SLE and non-LN groups, respectively (*p* = 0.000) (72.45 ± 23.60 versus 11.67 ± 6.89 for active versus inactive SLE; 78.97 ± 19.74 versus 10.56 ± 4.89 for LN versus non-LN) (Table 3). Serum concentrations of complements C3 and C4 were lower in patients with active SLE and LN relative to those with respective inactive SLE and non-LN groups (*p* < 0.05 for LN versus non-LN) (Table 3). Other autoantibodies, including antibodies to cardiolipin (ACL), perinuclear neutrophil cytoplasmics (pANCA), ribosomal P-proteins, ribonucleoprotein, and Sjögren's syndrome A and Sjögren's syndrome B, were also detected in SLE patients, which were listed in Table 3. Of note, significant difference in levels of anti-C1q and anti-dsDNA antibodies and complements C3 and C4 was found in patients with SLE as compared with those with a non-SLE autoimmune disease or healthy control cohorts (Table 4).

### 3.4. Correlations of Anti-C1q Antibodies with Anti-dsDNA Antibodies, Complement Levels, and SLEDAI Score

The correlation coefficients between the anti-C1q antibodies and anti-dsDNA antibodies, complement levels, and the SLEDAI score were summarized in Table 5. The titer of serum anti-C1q antibodies was positively correlated to anti-dsDNA antibodies (*r* = 0.796) and SLEDAI (*r* = 0.584) but inversely correlated to serum levels of complements C3 (*r* = −0.563) and C4 (*r* = −0.532) (Table 5). Of interest, the concentrations of antibodies to C1q and dsDNA correlated very well with each other (*r* = 0.796). Equally noteworthy, both autoantibodies correlated well with the SLEDAI score, despite the anti-C1q showed somewhat better with SLEDAI score than that of anti-dsDNA (*r* = 0.584 versus *r* = 0.475) (Table 5).

### 3.5. Significance of Anti-C1q Antibody for the Identification of Patients with Active SLE and LN

Higher frequency and titers of serum autoantibodies to C1q and dsDNA and lower levels of serum of the complement components C3 and C4 were detected in patients with active SLE and active nephritis compared to patients with inactive SLE and without a renal involvement, respectively (Tables 3 and 4). In order to evaluate the significance of anti-C1q antibodies in clinical settings, we analyzed the sensitivities and specificities of anti-C1q antibodies, anti-dsDNA antibodies, and levels of C3 and C4 alone or in a combination for the identification of patients with active SLE (Table 6) and LN (Table 7). Although both serum anti-dsDNA antibody and levels of complements (C3 and C4) alone displayed a superior sensitivity for identifying patients with active SLE and LN to anti-C1q antibodies, their specificities were inferior to anti-C1q antibody. Of note, a combination of anti-C1q antibodies and anti-dsDNA antibodies or serum levels of C3 and C4 could significantly increase specificities but decrease the sensitivities in identification of patients with active SLE and LN in comparison with these serological markers alone (*p* < 0.05) (Tables 6 and 7). Intriguingly, a combination of the three markers could not enhance the specificity further but reduced the sensitivity (Tables 6 and 7). The sensitivity, specificity, positive and negative predictive values (PPV and NPV), and odds ratio (OR) (95% CI) for identification of patients with active SLE were 76.5%, 90.9%, 90.7% and 76.9%, and 14.625 (5.365–39.846) for a combination of anti-C1q and anti-dsDNA antibodies, 78.4%, 95.5%, 95.2% and 79.2%, and 23.030 (7.749–68.451) for a combination of anti-C1q antibody and levels of C3 and C4, and 72.5%, 93.2%, 92.5% and 74.5%, and 26.429 (7.987–87.549) for a combination of anti-C1q antibody, anti-dsDNA antibody, and levels of C3 and C4 anti-C1q Ab (Table 6). The sensitivity, specificity, PPV, NPV, and OR (95% CI) for identification of patients with LN were 82.9%, 76.7%, 67.4%, 88.5%, and 11.278 (3.994–31.846) for a combination of anti-C1q and anti-dsDNA antibodies, 85.7%, 80.0%, 71.4%, 90.6%, and 16.500 (5.485–49.878) for a combination of anti-C1q antibody and levels of C3 and C4, and 80.0%, 70.0%, 76.0%, 80.4%, and 14.462 (5.157–40.553) for a combination of anti-C1q antibodies, anti-dsDNA antibodies, and levels of C3 and C4 anti-C1q Ab (Table 7). These data indicate that a combination of anti-C1q antibodies and other serological marker(s) may enhance the specificity in the identification of patients with active SLE and LN.

## 4. Discussion 

With increasing appreciation for the role of the complement system in processes of waste transport, immune tolerance, and shaping of the adaptive immune response, functional consequences of autoantibodies to complements have recently received a great deal of attention in the pathogenesis and diagnosis for autoimmune diseases. Among them, antibodies to C1q are one of the most interrogated serological markers in clinical settings, particularly in SLE disease [13, 31]. In this report, we evaluated the diagnostic value of serum anti-C1q alone or in combination with anti-dsDNA and/or serum levels of complements C3 and C4 for identification of patients with active SLE and LN in 95 SLE patients. The results showed that anti-C1q was more often and strikingly elevated in patients with active SLE and LN relative to patients with inactive SLE and non-LN, non-SLE autoimmune diseases, and healthy control individuals. In addition, the frequency and levels of anti-C1q antibodies were positively correlated with dsDNA antibodies and SLEDAI score but inversely correlated with levels of C3 and C4 in SLE patients. Of note, anti-C1q antibodies displayed a closer association with renal disease activity in SLE patients than anti-dsDNA antibodies and reduction of C3 and C4 in terms of diagnostic specificity. Combination of anti-C1q and antibodies to dsDNA and/or levels of C3 and C4 could enhance the specificity but reduce the sensitivity of these serological markers for identification of patients with active SLE diseases and LN. These data imply that anti-C1q may be a valuable diagnostic marker for patients with LN and active SLE diseases. Such observation is consistent with finding of anti-C1q from other groups [5, 10, 13, 15, 17, 22, 24, 37]. In addition, no difference in anti-C1q frequency between Chinese Han and Chinese Hui SLE patients was observed; this finding was in line with results from different countries and ethnic groups [5].

The immune dysfunction is a hallmark of SLE during its pathogenesis. The immune disorder is a main cause of productions of autoantibodies, deposition of immune complexes, and the defect of autoimmune tolerance, which in turn leads to injuries of multiple organs [2]. The complement system has been recognized to play crucial roles in scavenging immune complexes and autoantigens generated from cell apoptosis and prevent the autoimmune-induced tissue and organ injury [38]. Owing to its opsonizing role in the clearance of autoantigens and apoptotic bodies under a physiological condition, a defective function of complement system may lead to failure in properly identifying and promptly cleaning cell debris and autoantigens, which in turn activate immune response and produce autoantibodies. Sequentially, autoantibodies bind to complement fragments, which have a major functional consequence to cause tissue injury, especially in the kidney [39, 40]. This notion was supported by a finding in which a deficiency of C1q, a complement component involved in canonical activation pathway, could result in SLE-like diseases in humans [41].

Antibodies to C1q were initially identified as low molecular weight C1q precipitins in the sera of SLE patients [20], and its clinical association was also first made in SLE patients [42]. Antibodies to C1q were found to strongly associate with the development of LN, and SLE patients who do not have these antibodies were very unlikely to have active renal flares [3, 5, 10, 11, 37, 43, 44]. Since the involvement of renal flare in SLE diseases represents a major complication in the treatment, an early identification of LN would guide an early intervention for rheumatologists in a clinical setting.

It has been demonstrated that deficiencies of an early component (C1q, C1r, C1s, C2, and C4) of the canonical pathway of complement activation system were strongly correlated with the severity and progression in SLE [45]. Among these complements, C1q is the most extensively investigated and plays indispensable roles in the clearance of immune complexes and cell debris [10, 13, 15, 31]. In this context, an elevated anti-C1q may induce the formation of C1q-anti-C1q complexes and promote the production of inflammatory mediators, which in turn inhibit the activation of complement and the clearance of immune complexes, sequentially resulting in further release of autoantigens, production of autoantibodies, and formation of complexes, eventually activating diseases and leading to tissue damage [46]. Although the anti-C1q antibodies have recently gained increasing attention, owing to elevated anti-C1 antibodies often observed in the sera of patients with active SLE and LN, as well as other non-SLE diseases such as hypocomplementemic urticarial vasculitis, rheumatoid vasculitis, mixed connective tissue disease (MCTD), and IgA nephropathy, the clinical significance of anti-C1q antibodies in SLE activity and active LN has not been fully appreciated.

An increasing number of evidences suggest that serum anti-C1q antibodies are strongly associated with SLE activity, especially with SLE patients with active LN, which implies that anti-C1q antibody may be a valuable serological marker for identification of patients with active SLE disease and LN in clinical settings [3, 5, 9, 11, 12, 22]. The involvement of anti-C1q antibody in SLE activity and LN was validated in a murine model, in which mice injected with anti-C1q antibodies purified from SLE patients had proliferative glomerulonephritis and deposition of anti-C1q antibodies in glomeruli of mice [47], and mice deficient C1q showed SLE-like disease phenotype with production of anti-nuclear antibodies and glomerulonephritis [48, 49]. In agreement with these findings, we also observed a more frequent detection of anti-C1q antibodies in patients with active SLE and LN, in whom the circulating anti-C1q antibody was dramatically elevated in comparison with healthy cohorts, patients with non-SLE diseases, and those with inactive SLE and no renal involvement in this study, respectively (*p* < 0.001). This finding supports the view that anti-C1q antibodies can be used as an important diagnostic parameter for identifying SLE patients with active disease and LN [3, 5, 7, 10, 12, 17].

Despite the fact that anti-dsDNA antibodies have been used as a universally diagnostic criterion for SLE (70–98% of patients are positive for dsDNA antibodies) and for monitoring the SLE disease activity including the renal and central nervous involvements [7, 26, 50, 51], other autoantibodies such as anti-Sm antibodies [28, 51] and anti-nucleosome antibodies [25, 26, 28] are also excellent serological markers for SLE diagnosis and treatment monitoring, but their specificities in the identification of SLE disease activity and lupus flares are limited. Therefore, there is a necessity to include other marker(s) for improving the specificity for diagnosis of SLE activity and lupus flares, among which serum anti-C1q antibodies are one of the most attractive candidates, owing to its strong correlation with LN, the most common lupus flare in SLE, has been well established [5, 10, 12, 22, 46]. However, unlike its positive correlation with SLEDAI score, the association of anti-C1q antibodies with LN remains controversial in various studies [3, 5, 10–12, 16, 22, 46, 52, 53]. For example, Sinico et al. found that 44% of SLE patients had anti-C1q antibodies, among whom 60% were with LN and only 14% of SLE patients were without a renal flare, suggesting a correlation of anti-C1q with LN (*p* < 0.05) [22]. This finding was supported by a late study by Tan et al. using a large number of LN cohorts, in which the authors demonstrated that the presence of anti-C1q antibodies was associated with disease activity of SLE and lupus nephritis [17]. Such observation was further confirmed by recent study performed by Gargiulo Mde et al., who found that patients with positive anti-C1q antibodies had clinically active SLE disease, and the presence of anti-C1q was associated with LN activity [10]. Furthermore, they also observed that levels of anti-C1q were correlated with LN evolution during one year of follow-up analysis using the second time of serum samples, suggesting that increased levels of anti-C1q antibodies during the treatment could be associated with activity of LN and severity of renal histological lesion [10]. In addition, Moroni et al. tested a panel of autoantibodies and complements at the time of kidney biopsy and after treatment in 107 SLE patients with LN and found that anti-C1q was the only parameter correlated with the clinical presentation of LN [3]. However, controversies still exist in the field of SLE diseases. Several lines of discordant results were also reported; there was no overt correlation found between anti-C1q and renal flares in SLE patients, despite the fact that anti-C1q was positively associated with SLEDAI scores [54, 55]. For instance, Katsumata et al. recently demonstrated that antibodies to C1q were associated with global activity of SLE but not specifically with active LN [56]. The variability reported in different studies for anti-C1 frequencies could be in part attributed to differences in clinical features, types of nephropathy and the time of evolution of the course of disease, methodological differences in the commercial ELISA kits used, and immunogenetic polymorphisms [24]. For example, a strong association of anti-C1q antibodies and Fc*γ* receptor type IIA- (Fc*γ*RIIA-) H/R131 polymorphism in Caucasoid lupus patients was demonstrated [57]. In this study, Norsworthy et al. found a significantly increased frequency of the Fc*γ*RIIA-R131 allele in lupus patients with nephritis and the patients whom anti-C1q antibodies were detected in their sera, as compared with controls, suggesting that Fc*γ*RIIA-R131 was associated with production of anti-C1q antibodies and might be a risk factor for the development of SLE with manifestations in the kidney in Caucasoid patients [57]. In the present study, a strong correlation of the frequency and levels of anti-C1q antibodies with the activity of SLE disease and renal flares was also determined.

Previous studies have indicated that anti-dsDNA and anti-Sm antibodies are useful serological marker for identifying active SLE and LN activity [51]. However, different assays of anti-dsDNA antibodies have significant impacts on diagnosing SLE disease activity in terms of the sensitivity and specificity [24]. In addition to anti-dsDNA antibodies, reduction of complements C3 and C4 was also lacking specificity [24]. Julkunen et al. recently found that anti-C1q and complements C3 and C4 were better markers for LN activity than anti-dsDNA antibodies; on the other hand, anti-dsDNA antibodies and serum levels of C3 and C4 were better markers than anti-C1q for evaluating the overall and non-LN SLE activity [24]. Interestingly, strong correlations between the frequency of anti-C1q and anti-dsDNA antibodies and low levels of C3 and C4 were found in the present study, which were consistent with findings from other studies in SLE diseases [7, 24–26]. These studies and ours suggest that anti-C1q antibodies are a reliable serological marker for accessing the disease activity of SLE, especially the activity of renal flares.

In order to further evaluate the diagnostic value of anti-C1q antibodies in identifying activities of SLE and renal lesions, the sensitivity and specificity of anti-C1q alone and in combination with anti-dsDNA antibodies and/or levels of C3 and C4 were analyzed. The results implied that a combination of anti-C1q with anti-dsDNA antibodies and/or serum levels of C3 and C4 could significantly increase the specificity but decrease the sensitivity for identification of patients with active SLE diseases and LN in comparison with the use of each of these markers alone. This finding was in agreement with a previous observation in a study performed by Yang et al., in which the authors also stated that antibodies to C1q were more strongly correlated with the activity renal disease than anti-dsDNA antibodies, and a combination of anti-C1q with anti-dsDNA antibodies increased the specificity for identification of LN activity [7]. These studies further indicate that anti-C1q may be a valuable diagnostic and/or prognostic marker for SLE patients with active LN in a clinical setting.

## 5. Conclusions

In conclusion, this study in 95 SLE patients confirms previous findings of the correlation of anti-C1q antibodies with SLE disease activity and renal flares. Moreover, anti-C1q antibodies showed a stronger association with renal disease activity in SLE patients than anti-dsDNA antibodies and reduction of C3 and C4 in terms of diagnostic specificity. Importantly, a combination of anti-C1q and anti-dsDNA antibodies and/or serum levels of complements C3 and C4 leads to an increase of the specificity but decrease of the sensitivity for identification of SLE patients with active diseases and LN. This study thus supports a view that anti-C1q is a valuable serological marker for SLE disease activity and LN, which warrants further investigation in clinical settings. Limitations of this study include the fact that only a small number of 95 SLE samples were studied and follow-up data were also lacking and the LN activity was mainly determined by laboratory parameters and clinical manifestations rather than by pathogenic analysis in renal biopsies.

## Figures and Tables

**Table 1:** Demographics of patients with systemic lupus erythematosus (SLE) (*N* = 95) and non-SLE autoimmune diseases (*N* = 40) and healthy control cohorts (*N* = 34)^a^.

Demographics	SLE	Non-SLE	Healthy
Ethnics (Chinese Han/Hui)	95 (75/20)	40 (26/14)	34 (20/14)
Age (mean ± SD) (range, years old)	32.56 ± 11.37 (14–71)	33.56 ± 11.78 (15–69)	32.50 ± 4.87 (12–73)
Gender (male/female) (% female)	9/86 (90.5)	4/36 (90.0)	3/31 (91.2)
Disease duration (mean ± SD) (range, years)	5.32 ± 4.04 (0.2–18)	6.01 ± 3.79 (0.3–20)	NA
SLEDAI score (range)	12.03 ± 6.45 (0–35)	NA	NA

^a^Non-SLE (*N* = 40): other autoimmune diseases include rheumatoid arthritis (RA) (5 cases), primary Sjögren's syndrome (pSS) (10 cases), ankylosing spondylitis (AS) (2 cases), dermatomyositis (DM) (5 cases), Behçet's disease (BD) (4 cases), systemic sclerosis (SSc) (3 cases), undifferentiated connective tissue disease (UCTD) (5 cases), and systemic vasculitis (3 cases).

**Table 2:** The presence of anti-C1q antibodies in patients with SLE (mean ± SD) (*N* = 95).

	Active SLE^b^ (*N* = 51)			Inactive SLE (*N* = 44)		
	Anti-C1q positive	Anti-C1q negative	*p* value	Anti-C1q positive	Anti-C1q negative	*p* value
Patient number (%)	40/51 (78.4)	11/51 (21.6)	NA	10/44 (22.7)	34/44 (77.3)	NA
Gender (M/F) (% F)	4/37 (89.2)	2/9 (77.8)	NA	1/9 (88.9)	2/32 (93.8)	NA
Ages (years)	30.56 ± 8.21	30.89 ± 10.45	0.326	31.56 ± 11.37	34.44 ± 12.56	0.207
With LN number (%)	29/51 (56.9)	5/51 (9.8)	NA	1/44 (2.3)	0	NA

^b^Active SLE disease was defined by SLEDAI score greater than 10. LN: lupus nephritis; M/F: male/female.

**Table 3:** Association of the presence of laboratory parameters between active and inactive SLE with and without renal involvement (mean ± SD) (*N* = 95).

Group	Activity of SLE (*N* = 95)			SLE with renal involvement (*N* = 95)		
	Active SLE (*N* = 51)	Inactive SLE (*N* = 44)	*p* value	LN (*N* = 35)	Non-LN (*N* = 60)	*p* value
ACL Ab (+) number (%)	7/51 (13.7)	5/44 (11.4)	NA	4/35 (11.4)	8/60 (13.3)	NA
Anti-C1q (+) number (%)	40/51 (78.4)	10/44 (22.7)	NA	30/35 (85.7)	20/60 (33.3)	NA
Anti-dsDNA (+) number (%)	50/51 (98.0)	28/44 (63.6)	NA	34/35 (97.1)	44/60 (73.3)	NA
ANA (+) number (%)	51/51 (100)	44/44 (100)	NA	35/35 (100)	60/60 (100)	NA
Anti-Rib-P (+) number (%)	4/51 (7.8)	1/44 (2.3)	NA	3/35 (8.6)	2/60 (3.3)	NA
Anti-Smith (Sm) (+) number (%)	23/51 (45.1)	10/44 (22.7)	NA	19/35 (54.3)	14/60 (23.3)	NA
Anti-SSA Ab (+) number (%)	15/51 (29.4)	8/44 (18.2)	NA	12/35 (34.3)	11/60 (18.3)	NA
Anti-SSB Ab (+) number (%)	4/51 (7.8)	4/44 (2.3)	NA	4/35 (11.4)	4/60 (6.7)	NA
pANCA (+) number (%)	16/51 (31.4)	3/44 (6.8)	NA	8/35 (22.9)	11/60 (18.3)	NA
C3 (*μ*g/mL)	0.55 ± 0.16	0.97 ± 0.16	0.047	0.47 ± 0.22	1.09 ± 0.16	0.031
C4 (*μ*g/mL)	0.14 ± 0.05	0.17 ± 0.07	0.142	0.12 ± 0.04	0.19 ± 0.10	0.045
Anti-C1q (AU/mL)	60.9 ± 11.6	8.3 ± 3.5	0.000	68.1 ± 14.6	14.1 ± 3.8	0.000
Anti-dsNDA (IU/mL)	72.45 ± 23.60	11.67 ± 6.89	0.000	78.97 ± 19.74	10.56 ± 4.89	0.000
Hemoglobin (*μ*g/mL)	24.6 ± 11.7	12.7 ± 0.89	0.008	25.7 ± 9.8	11.2 ± 1.92	0.004
Urine protein (g/24 hours)	1.19 ± 0.61	0.11 ± 0.04	0.003	1.31 ± 0.77	0.09 ± 0.07	0.000

Ab: antibody; ACL: anti-cardiolipin; ANA: anti-nuclear antibody; LN: lupus nephritis; pANCA: perinuclear anti-neutrophil cytoplasmic antibody; Rib-P: ribosomal P-proteins; RNP: ribonucleoprotein; SSA: Sjögren's syndrome A; SSB: anti-Sjögren's syndrome B.

**Table 4:** Significant difference in levels of anti-C1q and anti-dsDNA antibodies and C3 and C4 in patients with SLE compared to those with non-SLE autoimmune diseases or healthy individuals.

Group (*N*)	Anti-C1q		Anti-dsDNA		Complements C3 and C4 (mean ± SD)		
	Positive *n* (%)	Concentration (mean ± SD) (AU/mL)	Positive *n* (%)	Concentration (mean ± SD) (IU/mL)	Low C3 and C4 *n* (%)^c^	C3 (*μ*g/mL)	C4 (*μ*g/mL)
SLE (95)	50 (52.6)	106.8 ± 17.5	78 (82.1)	67.9 ± 20.2	61 (64.2)	0.57 ± 0.19	0.16 ± 0.08
Non-SLE (40)^a^	3 (7.5)	15.7 ± 10.4^b^	4 (10)	5.7 ± 1.6^b^	3 (7.5)	1.25 ± 0.24^b^	0.29 ± 0.08^b^
Healthy (34)	1 (2.9)	8.5	2 (5.9)	4.8 ± 1.7	1 (2.9)	1.34	0.28

^a^Non-SLE (*N* = 40): other autoimmune diseases include rheumatoid arthritis (RA) (5 cases), primary Sjögren's syndrome (pSS) (10 cases), ankylosing spondylitis (AS) (2 cases), dermatomyositis (DM) (5 cases), Behçet's disease (BD) (4 cases), systemic sclerosis (SSc) (3 cases), undifferentiated connective tissue disease (UCTD) (5 cases), and systemic vasculitis (3 cases). ^b^Compared to SLE group, *p* < 0.05; ^c^C3 and C4 below normal range.

**Table 5:** Correlation coefficients between anti-C1q antibodies, anti-dsDNA antibodies, C3, C4, and SLEDAI score.

	Anti-C1q	Anti-dsDNA	C3	C4	SLEDAI score
Anti-C1q	1.000				
Anti-dsDNA	0.796	1.000			
C3	−0.563	−0.627	1.000		
C4	−0.532	−0.416	0.627	1.000	
SLEDAI score	0.584	0.475	−0.311	−0.378	1.000

**Table 6:** Significant differences in concentration, sensitivity, specificity, and odds ratio (OR) based on disease activity in SLE patients (*N* = 95)^a^.

	Sensitivity (%)	Specificity (%)	PPV (%)	NPV (%)	OR (95% CI)
Anti-C1q Ab	40/51 (78.4)	34/44 (77.3)	40/50 (80.0)	34/45 (75.6)	12.364 (4.683–32.640)
Anti-dsDNA Abs	50/51 (98.0)	16/44 (36.4)	50/78 (64.1)	16/17 (94.1)	28.571 (3.596–227.008)
Levels of C3 and C4	46/51 (90.2)	29/44 (65.9)	46/61 (75.4)	29/34 (85.3)	17.787 (5.840–54.172)
Anti-dsDNA Ab, levels of C3 and C4	45/51 (88.2)	24/44 (54.5)	45/65 (69.2)	24/30 (80.0)	17.885 (6.134–52.143)
Abs to C1q and dsDNA	39/51 (76.5)	40/44 (90.9)	39/43 (90.7)	40/52 (76.9)	14.625 (5.365–39.846)
Anti-C1q Ab, levels of C3 and C4	40/51 (78.4)	42/44 (95.5)	40/42 (95.2)	42/53 (79.2)	23.030 (7.749–68.451)
Abs to C1q and dsDNA, levels of C3 and C4	37/51 (72.5)	41/44 (93.2)	37/40 (92.5)	41/55 (74.5)	26.429 (7.987–87.549)

^a^The sensitivity, specificity, and predictive values were calculated using formula described in a previous report [27]. PPV: positive predictive value; NPV: negative predictive value.

**Table 7:** Significant differences in concentration, sensitivity, specificity, and odds ratio (OR) based on renal involvement in SLE patients (*N* = 95)^a^.

	Sensitivity (%)	Specificity (%)	PPV (%)	NPV (%)	OR (95% CI)
Anti-C1q Ab	30/35 (85.7)	40/60 (66.7)	30/50 (60.0)	40/45 (88.9)	12.000 (4.041−35.632)
Anti-dsDNA Ab	34/35 (97.1)	16/60 (26.7)	34/78 (43.6)	16/17 (94.1)	12.364 (1.561−97.907)
Levels of C3 and C4	34/35 (97.1)	33/60 (55.0)	34/61 (55.7)	33/34 (97.1)	41.556 (5.336−323.637)
Anti-dsDNA Ab, levels of C3 and C4	33/35 (94.3)	31/60 (51.7)	33/62 (53.2)	31/33 (93.9)	23.100 (5.069−105.276)
Abs to C1q and dsDNA	29/35 (82.9)	46/60 (76.7)	29/43 (67.4)	46/52 (88.5)	11.278 (3.994−31.846)
Anti-C1q Ab, levels of C3 and C4	30/35 (85.7)	48/60 (80.0)	30/42 (71.4)	48/53 (90.6)	16.500 (5.485−49.878)
Abs to C1q and dsDNA, levels of C3 and C4	29/35 (80.0)	42/60 (70.0)	29/47 (76.0)	38/48 (80.4)	14.462 (5.157−40.553)

^a^The sensitivity, specificity, and predictive values were calculated using formula described in a previous report [27]. PPV: positive predictive value; NPV: negative predictive value.

## Abbreviations

Ab: Antibody
ACR: American College of Rheumatology
ACL: Anti-cardiolipin
ANA: Anti-nuclear antibody
AS: Ankylosing spondylitis
BD: Behçet's disease
C1q: Complement 1q
DM: Dermatomyositis
dsDNA: Double-stranded DNA
HUVS: Hypocomplementemic urticarial vasculitis syndrome
LN: Lupus nephritis
MCTD: Mixed connective tissue disease
pANCA: Perinuclear anti-neutrophil cytoplasmic antibody
pSS: Primary Sjögren's syndrome
RA: Rheumatoid arthritis
Rib-P: Ribosomal P-proteins
RNP: Ribonucleoprotein
SLE: Systemic lupus erythematosus
SSA: Sjögren's syndrome A
SSB: Sjögren's syndrome B
SSc: Systemic sclerosis
UCTD: Undifferentiated connective tissue disease.
